# Detecting influential subjects in intensive longitudinal data using mixed-effects location scale models

**DOI:** 10.1186/s12874-023-02046-9

**Published:** 2023-10-18

**Authors:** Xingruo Zhang, Donald Hedeker

**Affiliations:** Department of Public Health Sciences, The University of Chicago, 5841 South Maryland Ave MC2000, Chicago, 60637 IL USA

**Keywords:** Cook’s distance, Influential data, Intensive longitudinal data, Mixed-effects location scale model, Variance modeling

## Abstract

**Background:**

Collection of intensive longitudinal health outcomes allows joint modeling of their mean (location) and variability (scale). Focusing on the location of the outcome, measures to detect influential subjects in longitudinal data using standard mixed-effects regression models (MRMs) have been widely discussed. However, no existing approach enables the detection of subjects that heavily influence the scale of the outcome.

**Methods:**

We propose applying mixed-effects location scale (MELS) modeling combined with commonly used influence measures such as Cook’s distance and DFBETAS to fill this gap. In this paper, we provide a framework for researchers to follow when trying to detect influential subjects for both the scale and location of the outcome. The framework allows detailed examination of each subject’s influence on model fit as well as point estimates and precision of coefficients in different components of a MELS model.

**Results:**

We simulated two common scenarios in longitudinal healthcare studies and found that influence measures in our framework successfully capture influential subjects over 99% of the time. We also re-analyzed data from a health behavior study and found 4 particularly influential subjects, among which two cannot be detected by influence analyses via regular MRMs.

**Conclusion:**

The proposed framework can help researchers detect influential subject(s) that will be otherwise overlooked by influential analysis using regular MRMs and analyze all data in one model despite influential subjects.

**Supplementary Information:**

The online version contains supplementary material available at 10.1186/s12874-023-02046-9.

## Background

In the past decades, intra-individual variability, also called within-subject (WS) heterogeneity or level-1 heterogeneity, of health behaviors and conditions has received increased attention [1–3]. Accordingly, new developments in statistical modeling, including mixed-effects location scale (MELS) models proposed by Hedeker et al. [4] and Nordgren et al. [5], allow researchers to model the mean, or location, and variability, or (square of the) scale of responses simultaneously.

Although these models can accommodate between-subject (BS) heterogeneity, also called level-2 heterogeneity, through random subject effects, there often exist subjects who are so different from the others that they need additional analyses and/or may affect model estimation. For example, in ecological momentary assessments (EMA) studies, a typical type of careless responses is that subjects give many consecutive items the same answer [6]. In this case, even if the data from these subjects do not deviate from any pattern in mean responses that subjects correctly responding to questions may show, they exhibit exceptionally low variability. Such abnormal behavior can distort the parameter estimates, especially the estimated variance of random scale effects. On the other hand, subjects who display exceptional consistency during behavioral studies, such as a study that aims at increasing subjects’ physical activities, might be of interest as such a pattern may indicate good adherence to the study protocol. Being able to identify such subjects is important in terms of informing personalized healthcare. Especially, there have been discussions about using mixed-effects regression models (MRMs) to find individuals who behave differently from others regarding their health conditions and outcomes [7, 8]. Since MELS models are an extension of standard MRMs, they can also perform similar functions and offer additional information about WS variability.

While there has been extensive literature discussing influential subjects and observations in MRMs [7, 9, 10] and software developed to implement these methods [11], to the best of our knowledge, there lack similar methods for the scale model of the response. As we will show in later sections, not considering WS variability in influence analysis can leave subject(s) with abnormal WS variability undetected. Hence, in the topic of influence analysis, WS variability deserves equal attention as the mean of the outcomes.

Therefore, we propose a framework for detecting influential subjects using MELS models. We examine each subject’s influence on model fit as well as point estimates of parameters and their efficiency. The proposed method enables researchers to identify subjects who exhibit dubious patterns in their WS variability and/or mean, thus facilitating research on intra-individual variability. Unlike the traditional case-deletion approach of influential data detection, we adopt the method proposed by Langford and Lewis [9]. If a subject is being examined for its influence, it is removed from the estimation of level-2 effects and given subject-specific fixed effects. We will demonstrate how the estimation of the leave-one-out models can be carried out in SAS and R. Also, a health behavior study will be used as an example to illustrate the proposed methodology. Finally, we will assess the performance of our method and demonstrate its benefits over influence analysis using MRMs via simulated examples.

## Methods

### Leave-one-out MELS model

In this section, we first briefly review MELS models developed by Hedeker et al. [4]. For simplicity, only random intercepts with scalar variances are included in both the location and the scale models described below. Nevertheless, more complicated models that include random slopes of time-varying covariates and/or have covariates influence the variances of the random effects are possible.

Suppose that subject *i* ($i = 1, 2, \dots , N$) is measured at visit *j* ($j = 1, 2, \dots , n_i$). The model for the response $y_{ij}$ can be expressed as:

1 $$\begin{aligned} y_{ij} = \beta _0 + \nu _i + x^T_{ij}\beta ' + \epsilon _{ij}, \end{aligned}$$

where $x_{ij}$ is a $p\times 1$ vector of time-varying covariates influencing the mean of $y_{ij}$, and $\nu _i$ is subject *i*’s random intercept, indicating subject *i*’s deviation from the fixed part of the model. $\beta _0$ is the fixed intercept, i.e. average response when all covariates equal 0, and $\beta '$ is a $p \times 1$ vector of coefficients corresponding to $x_{ij}$. $\epsilon _{ij}$ is the level-1 residual and follows a normal distribution with a mean of 0 and a variance of $\sigma ^2_{\epsilon _{ij}}$. Later on, the notation $\beta$ in influence measures represents a vector of which the first entry is $\beta _0$ and the remaining entries come from $\beta '$.

In the MELS model, a log-linear sub-model is applied to the WS variance of the response to ensure a positive value:

2 $$\begin{aligned} \sigma ^2_{\epsilon _{ij}} = \textrm{exp}\left( \tau _0 + \omega _i+ w^T_{ij}\tau '\right) , \end{aligned}$$

where $w_{ij}$ is an $r \times 1$ vector of time-varying covariates influencing the scale of $y_{ij}$, and $\omega _i$ is the $i^{th}$ subject’s deviation from the average log WS variance of $y_{ij}$. $\tau _0$ is the average log WS variance when every covariate in $w_{ij}$ is 0, and $\tau '$ is an $r \times 1$ vector of coefficients of $w_{ij}$. Likewise, $\tau$ in influence measures refers to a vector of which the first entry is $\tau _0$, and the remaining entries come from $\tau '$.

The random location and scale effects can correlate with each other, and they are assumed to follow a bivariate normal distribution:

3 $$\begin{aligned} \left( \begin{array}{c} \nu _i \\ \omega _i \end{array}\right) \sim \mathcal {N}\left( \left( \begin{array}{c} 0 \\ 0 \end{array}\right) , \left( \begin{array}{cc} \sigma ^2_{\nu } &{} \sigma _{\nu \omega } \\ \sigma _{\nu \omega } &{} \sigma ^2_{\omega } \end{array}\right) \right) . \end{aligned}$$

Given our focus on influence analysis at the subject level, also known as level 2 in longitudinal models, it’s important to pause here and clarify that level-2 effects in MELS models comprise fixed effects of time-invariant covariates (including the fixed intercepts) and all random effects. This distinction lays the foundation for our forthcoming exploration of influence analysis using MELS models. The following paragraphs present how the leave-one-out MELS models are formed, i.e., how each subject is separated from the random effects and subject-level fixed effects and given subject-specific fixed effects, for subsequent influence analyses.

As suggested by Langford and Lewis [9], in order to separate the subject under evaluation, denoted as $i^*$ $(i^* \in 1, 2, \dots , N$), from level 2 of the location model, we exclude subject $i^*$ from the estimation of the random effects and level-2 fixed effects. In this case, the only level-2 fixed effect is $\beta _0$. Then, subject $i^*$ is given a subject-specific fixed effect denoted as $c_{i^*}$. Namely, the location model becomes

4 $$\begin{aligned} y_{ij} = \mathbbm {1}(i \ne i^*)\times (\beta _{0(-i^*)} +\nu _{i}) + x^T_{ij}\beta '_{(-i^*)} + \mathbbm {1}(i = i^*)\times c_{i^*} + \epsilon _{ij}, \end{aligned}$$

where $\mathbbm {1}(i = i^*)$ equals 1 for subject $i^*$ and 0 for all other subjects, and $\mathbbm {1}(i \ne i^*)$ equals 0 for subject $i^*$ and 1 for all other subjects.

Note that for simplicity in illustrating leave-one-out MELS models, we assume that every covariate in $x_{ij}$ and $w_{ij}$ is time-varying. When some time-invariant covariates are also present, subject $i^*$ should be separated from the estimation of their associated coefficients as well.

To also separate subject $i^*$ from level 2 of the scale model described in Eq. 2, the leave-one-out scale model is as follows:

5 $$\begin{aligned} \sigma ^2_{\epsilon _{ij}} = \textrm{exp}(\mathbbm {1}(i \ne i^*)\times (\tau _{0(-i^*)} + \omega _{i}) + w^T_{ij}\tau '_{(-i^*)} + \mathbbm {1}(i = i^*)\times d_{i^*}). \end{aligned}$$

Here, $d_{i^*}$ is the $i^{*th}$ subject’s subject-specific fixed scale effect, and the variances of random location effects and random scale effects become $\sigma ^2_{\nu (-i^*)}$ and $\sigma ^2_{\omega (-i^*)}$ while their covariance is $\sigma _{\nu \omega (-i^*)}$ without subject $i^*$. Note that the subscript $(-i^*)$ here as well as in Eqs. 4 and 5 does not mean that subject $i^*$ is entirely removed from modeling but instead only removed from the estimation of level-2 effects.

Overall, the algorithm to estimate a leave-one-out MELS model includes the following steps: (1) create an indicator variable that equals 1 for subject $i^*$ and 0 otherwise; (2) use the opposite of the indicator variable in (1) as the covariate for the random intercepts; (3) following step (2), subject $i^*$ will not be included in the random effect estimation, and the fixed location effect for the indicator variable would be $c_{i^*}$ while the fixed scale effect is $d_{i^*}$. Our programming specifics and pertinent material will be explained in Section “Results”.

A separate leave-one-out model is estimated for every subject and compared with the model in which all subjects are treated the same. Besides allowing the detection of highly influential subjects, this leave-one-out structure can be viewed as a way to keep all subjects in the model despite influential subject(s). Using all available data to estimate the level-1 fixed effects has the benefit of increasing statistical power.

### Influence analysis

#### Influence on model fit

Given that the model described in Eqs. 1 and 2 are nested in the leave-one-out model described in Eqs. 4 and 5, likelihood-ratio test was used to examine subject $i^*$’s influence on the model fit. The test statistic, difference in deviance, denoted as $LR_{i^*}$ for subject $i^*$, can be calculated as follows:

6 $$\begin{aligned} LR_{i^*} = 2\ln \left( \frac{\mathcal {L}_{(-i^*)}}{\mathcal {L}}\right) , \end{aligned}$$

where $\mathcal {L}_{(-i^*)}$ represents the likelihood of the MELS model in which subject $i^*$ is separated, and $\mathcal {L}$ represents the likelihood of the naive MELS model. Since N tests are conducted simultaneously, the false discovery rate (FDR) procedure [12] is applied to adjust for multiple comparisons. This specific multiple testing correction method is chosen because of its ability to control for both Type I and Type II errors [13].

#### Influence on parameter estimates

Cook’s distance [14] is a well-known measure for the influence of data on the point estimates of a group of parameter estimates. Based on the structure of MELS models, we calculate the Cook’s distances for three groups of parameters, namely, fixed location effects ($\beta$), fixed scale effects ($\tau$), and variances and covariance of random effects, denoted as $\eta$ ($\eta = [\sigma ^2_{\nu }, \sigma ^2_{\omega }, \sigma _{\nu \omega }]$). Following the formula of Cook’s distance for multilevel models described by Snijder and Bosker [15], Cook’s distance can be calculated as

7 $$\begin{aligned} C_{i^*}^{\gamma } = \frac{1}{r_{\gamma }}\left(\hat{\gamma } - \hat{\gamma }_{(-i^*)}\right)^T\hat{\Sigma }_{\hat{\gamma }(-i^*)}^{-1}\left(\hat{\gamma } - \hat{\gamma }_{(-i^*)}\right), \end{aligned}$$

where $\gamma$ can be $\beta$, $\tau$, or $\eta$. Here, $r_{\gamma }$ is the number of parameters being examined, and $\Sigma _{\hat{\gamma }(-i^*)}$ is the variance-covariance matrix of $\hat{\gamma }$ after subject $i^*$ is separated from the random effect estimation. A large Cook’s distance indicates a heavy influence on a specific group of parameter estimates.

Once a subject is determined to be influential on a particular group of parameter estimates, it is often of interest to investigate this subject’s influence on each specific parameter estimate within this group. In this case, DFBETAS can be used. DFBETAS is the difference between the estimate of a parameter obtained when all subjects are kept in the random effect and level-2 effect estimation and the parameter estimate when a subject is separated, divided by the standard error of the parameter estimate [16]. Its mathematical representation is as follows:

8 $$\begin{aligned} \text {DFBETAS}_{i^*}^{\theta } = \frac{\hat{\theta } - \hat{\theta }_{(-i^*)}}{SE\left(\hat{\theta }_{(-i^*)}\right)}, \end{aligned}$$

where $\theta$ can be any single parameter in the model. DFBETAS can have both positive and negative values. A highly positive value indicates that the inclusion of the influential subject creates a positive bias for $\hat{\theta }$, and vice versa.

#### Influence on the precision of parameter estimates

Influential subjects can also affect the variances of parameter estimates besides their point estimates. COVTRACE proposed by Christensen et al. [10] and COVRATIO described by Belsley et al. [17] are often used to measure such changes.

$\text {COVTRACE}^{\gamma }_{i^*}$ is calculated as the absolute value of the difference between the trace of the inverse ratio of variance-covariance matrix estimates with and without subject $i^*$ in level-2 model and the number of parameters under investigation:

9 $$\begin{aligned} {\text{COVTRACE}}_{i^*}^{\gamma } = \left| {\textrm{Tr}}\left(\hat{\Sigma }_{\hat{\gamma }}^{-1}\hat{\Sigma }_{\hat{\gamma }(-i^*)}\right) - r_{\gamma }\right|. \end{aligned}$$

The larger the COVTRACE value, the greater the influence of subject $i^*$ on the precision of $\hat{\gamma }$.

Meanwhile, $\text {COVRATIO}^{\gamma }_{i^*}$ is the inverse ratio of the determinant of the estimated variance-covariance matrix of $\gamma$ with and without subject $i^*$ in level-2 model:

10 $$\begin{aligned} \text {COVRATIO}_{i^*}^{\gamma } = \frac{\det \left(\hat{\Sigma}_{\hat{\gamma }(-i^*)}\right)}{\det (\hat{\Sigma }_{\hat{\gamma }})}. \end{aligned}$$

Again, $\gamma$ is one of $\beta$, $\tau$, and $\eta$. The determinant of a variance-covariance matrix is also known as the generalized variance, a measure of multidimensional scatter [18]. The examples in the next two sections will focus on COVRATIO because it provides information on both the magnitude and the direction of the influence. A value of COVRATIO above 1 indicates a loss in precision when separating subject $i^*$, and on the contrary, a value below 1 indicates a gain in precision.

All the procedures of influence analysis described in Section “Influence analysis” are summarized in Table 1.

**Table 1:** Influence analysis framework for MELS models

Influence measure	Influence sub-category	Formula
Influence on model fit		
Difference in deviance		$LR_{i^*} = 2\ln \left( \frac{\mathcal {L}_{(-i^*)}}{\mathcal {L}}\right)$
Influence on point estimates of a group of parameters		
Cook’s distance	Fixed location effect estimates	$C_{i^*}^{\beta } = \frac{1}{r_{\beta }}\left(\hat{\beta } - \hat{\beta }_{(-i^*)}\right)^T{\hat{\Sigma }}_{\hat{\beta }(-i^*)}^{-1}\left(\hat{\beta } - \hat{\beta }_{(-i^*)}\right)$
	Fixed scale effect estimates	$C_{i^*}^{\tau } = \frac{1}{r_{\tau }}\left(\hat{\tau } - \hat{\tau }_{(-i^*)}\right)^T\hat{\Sigma }_{\hat{\tau }(-i^*)}^{-1}\left(\hat{\tau } - \hat{\tau }_{(-i^*)}\right)$
	Variances and covariances of random effects	$C_{i^*}^{\eta } = \frac{1}{r_{\eta }}\left(\hat{\eta } - \hat{\eta }_{(-i^*)}\right)^T\hat{\Sigma }_{\hat{\eta }(-i^*)}^{-1}\left(\hat{\eta } - \hat{\eta }_{(-i^*)}\right)$
Influence on point estimate of a single parameter		
DFBETAS		$\text {DFBETAS}_{i^*}^{\theta } = \frac{\hat{\theta } - \hat{\theta }_{(-i^*)}}{SE\left(\hat{\theta }_{(-i^*)}\right)}$
Influence on variances and covariances of a group of parameters		
COVTRACE	Fixed location effect estimates	${\text {COVTRACE}}_{i^*}^{\beta } = \left|{\textrm{Tr}}\left({\hat{\Sigma }}_{{\hat{\beta }}}^{-1}{\hat{\Sigma }}_{{\hat{\beta }}(-i^*)}\right) - r_{\beta }\right|$
	Fixed scale effect estimates	${\text {COVTRACE}}_{i^*}^{\tau } = \left|{\textrm{Tr}}\left(\hat{\Sigma }_{\hat{\tau }}^{-1}\hat{\Sigma }_{\hat{\tau }(-i^*)}\right) - r_{\tau }\right|$
	Variances and covariances of random effects	${\text {COVTRACE}}_{i^*}^{\eta } = \left|{\textrm{Tr}}\left(\hat{\Sigma }_{\hat{\eta }}^{-1}\hat{\Sigma }_{\hat{\eta }(-i^*)}\right) - r_{\eta }\right|$
COVRATIO	Fixed location effect estimates	${\text {COVRATIO}}_{i^*}^{\beta } = \frac{\det \left(\hat{\Sigma }_{\hat{\beta }(-i^*)}\right)}{\det \left(\hat{\Sigma }_{\hat{\beta }}\right)}$
	Fixed scale effect estimates	${\text{COVRATIO}}_{i^*}^{\tau } = \frac{\det \left(\hat{\Sigma }_{\hat{\tau }(-i^*)}\right)}{\det \left(\hat{\Sigma }_{\hat{\tau }}\right)}$
	Variances and covariances of random effects	${\text{COVRATIO}}_{i^*}^{\eta } = \frac{\det \left(\hat{\Sigma }_{\hat{\eta }(-i^*)}\right)}{\det \left(\hat{\Sigma }_{\hat{\eta }}\right)}$

## Results

### Application to health behavior study example

Data collected by Flueckiger et al. [19] for a study on the association between health behaviors and learning goal achievements are used as an example to illustrate the proposed method.

During the 32-day span of the study, 72 students answered questions about their sleep quality (SQ), physical activity, positive and negative affect, learning goal achievement (LGA), and examination grades. The following analysis focuses on complete observations with values for all variables, so our analysis includes 1788 observations from 62 subjects. One of the major findings from the original study is that better SQ is positively associated with LGA. Hence, we chose SQ as the covariate for both the mean and the scale models. Namely, the location model for the example can be expressed as

11 $$\begin{aligned} \text {LGA}_{ij} = \beta _0 + \nu _i + \beta _{\text {SQ}}\text {SQ}_{ij} + \epsilon _{ij}, \end{aligned}$$

and the scale model, in which the variance of $\epsilon _{ij}$ is being modeled, is

12 $$\begin{aligned} \sigma ^2_{\epsilon _{ij}} = \textrm{exp}(\tau _0 + \omega _i + \tau _{\text {SQ}}\text {SQ}_{ij} ), \end{aligned}$$

where SQ was measured on a 4-point Likert scale in which 1 means very bad, and 4 means very good. LGA was measured on a 5-point Likert scale in which 0 represents having not achieved the goals at all and 4 represents having achieved the goals completely. We treated LGA and SQ as continuous, consistent with the approach taken in the original study. The approximate normal distribution of LGA has been validated through our exploratory analysis. Nevertheless, it is important for the readers to exercise caution when generalizing the results beyond the original LGA range.

**Fig. 1:**
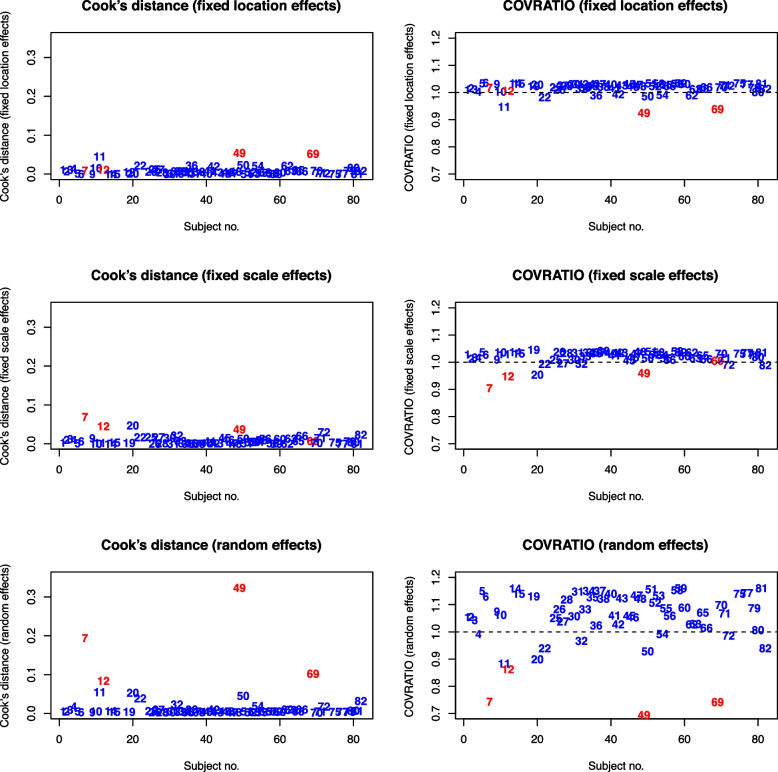
Cook’s distances and COVRATIOs for the health behavior data. Points in red are the four subjects influential on the fit of the MELS model of health behavior data

The Cook’s distances and COVRATIOs of all subjects are visualized in Fig. 1. After applying the FDR procedure, subjects 7, 12, 49, and 69 are determined to have a statistically significant influence on the model fit based on likelihood-ratio tests. Influence analysis results of these four subjects are summarized in Table 2. According to the Cook’s distances, we can see that subjects 7 and 12 have high influence on the fixed scale effects, subject 69 has high influence on the fixed location effects, while subject 49 has high influence on both. In particular, subject 7 has a large DFBETAS for $\tau _{\text {SQ}}$, subject 49 has the largest DFBETAS for $\beta _0$ and the smallest DFBETAS for $\tau _0$, and subject 69 has the smallest DFBETAS for $\beta _0$ and the $2^{nd}$ largest DFBETAS for $\beta _{\text {SQ}}$. All four subjects have high influence on the point estimates of the variances and covariance of the random effects. Specifically, separation of subjects 7, 12, and 49 shrinks $\hat{\sigma }^2_{\omega }$; separation of subjects 49 and 69 shrinks $\hat{\sigma }^2_{\nu }$; separation of subject 7 and 69 decreases $\hat{\sigma }_{\nu \omega }$ while separation of subject 12 and 49 increases $\hat{\sigma }_{\nu \omega }$. Moreover, excluding each of these subjects from the random effect estimation also shrinks the corresponding generalized variances of the groups of parameters that they have influence on the point estimates, suggesting that these four subjects have caused a loss in model precision in the original model.

**Table 2:** Influence analysis results for health behavior data

Subject	Influence measure	Results
7	Cook’s distance	- Largest $C^{\tau }$ (0.069)
		- $2^{nd}$ largest $C^{\eta }$ (0.194)
	DFBETAS	- $3^{rd}$ largest $\text {DFBETAS}^{\beta _{SQ}}$ (0.055)
		- Largest $\text {DFBETAS}^{\tau _{SQ}}$ (0.163)
		- Largest $\text {DFBETAS}^{\sigma ^2_{\omega }}$ (0.604)
		- Largest $\text {DFBETAS}^{\sigma _{\nu \omega }}$ (0.325)
	COVRATIO	- Smallest $\text {COVRATIO}^{\tau }$ (0.905)
		- $3^{rd}$ smallest $\text {COVRATIO}^{\eta }$ (0.743)
12	Cook’s distance	- $3^{rd}$ largest $C^{\tau }$ (0.045)
		- $4^{th}$ largest $C^{\eta }$ (0.084)
	DFBETAS	- $4^{th}$ largest $\text {DFBETAS}^ {\beta _0}$ (0.096)
		- $2^{nd}$ largest $\text {DFBETAS}^{\sigma ^2_{\omega }}$ (0.392)
		- $2^{nd}$ smallest $\text {DFBETAS}^{\sigma _{\nu \omega }}$ (-0.343)
	COVRATIO	- $2^{nd}$ smallest $\text {COVRATIO}^{\tau }$ (0.948)
		- $4^{th}$ smallest $\text {COVRATIO}^{\eta }$ (0.862)
49	Cook’s distance	- Largest $C^{\beta }$ (0.054)
		- $4^{th}$ largest $C^{\tau }$ (0.037)
		- Largest $C^{\eta }$ (0.323)
	DFBETAS	- Largest $\text {DFBETAS}^{\beta _0}$ (0.194)
		- Smallest $\text {DFBETAS}^{\tau _0}$ (-0.140)
		- Largest $\text {DFBETAS}^{ \sigma ^2_{\nu }}$ (0.553)
		- $4^{th}$ largest $\text {DFBETAS}^{\sigma ^2_{\omega }}$ (0.325)
		- Smallest $\text {DFBETAS}^{\sigma _{\nu \omega }}$ (-0.737)
	COVRATIO	- Smallest $\text {COVRATIO}^{\beta }$ (0.925)
		- $4^{th}$ smallest $\text {COVRATIO}^{\tau }$ (0.959)
		- Smallest $\text {COVRATIO}^{\eta }$ (0.694)
69	Cook’s distance	- $2^{nd}$ largest $C^{\beta }$ (0.052)
		- $3^{rd}$ largest $C^{\eta }$ (0.102)
	DFBETAS	- Smallest $\text {DFBETAS}^{\beta _0}$ (-0.281)
		- $2^{nd}$ largest $\text {DFBETAS}^{\beta _{SQ}}$ (0.121)
		- $2^{nd}$ largest $\text {DFBETAS}^{\sigma ^2_{\nu }}$ (0.422)
		- $3^{rd}$ largest $\text {DFBETAS}^{\sigma _{\nu \omega }}$ (0.269)
	COVRATIO	- $2^{nd}$ smallest $\text {COVRATIO}^{\beta }$ (0.939)
		- $2^{nd}$ smallest $\text {COVRATIO}^{\eta }$ (0.741)

**Fig. 2:**
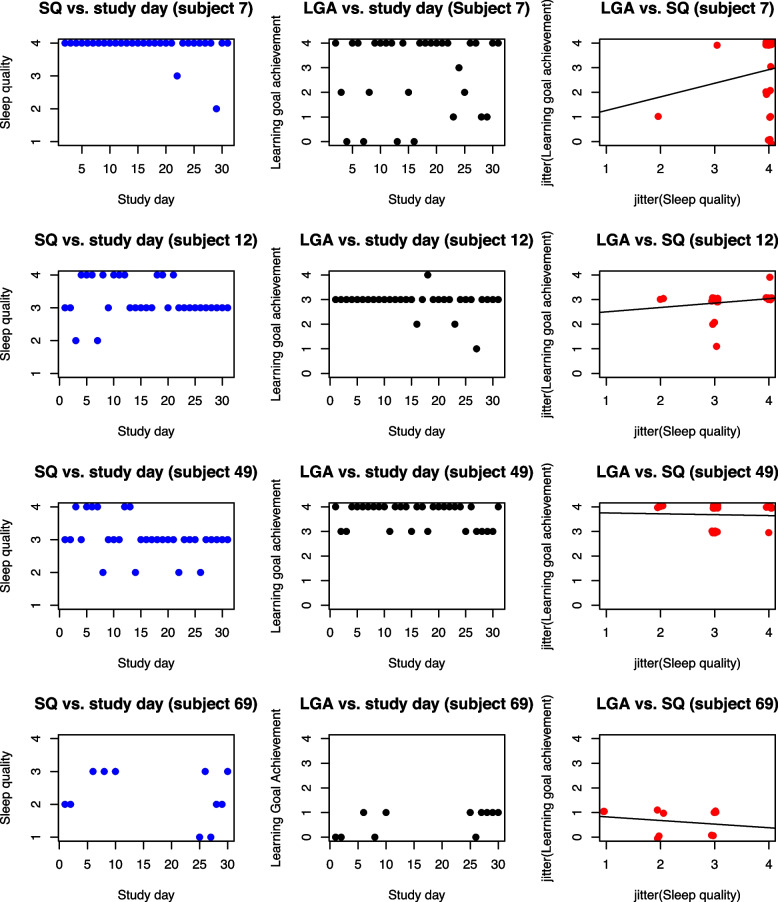
Data from subjects influential on the fit of the MELS model of health behavior data

We visualize the SQ and LGA of these four subjects in Fig. 2 to see how the data correspond with the influence analysis findings. While all except two of subject 7’s self-reported SQ ratings have a value of 4, the LGA of this subject was highly variable when SQ equaled 4. This is consistent with the influence analysis result that giving subject 7 subject-specific fixed effects shrinks $\hat{\tau }_{\text {SQ}}$ and $\hat{\sigma }^2_{\omega }$. While subject 49 had consistently high LGA, subject 69 had all low LGA values at 0 and 1. Such patterns in the data correspond to subject 49’s large $\text {DFBETAS}_{\beta _0}$ and subject 69’s small $\text {DFBETAS}_{\beta _0}$. Given subject 12, 49, and 69 have such great influence on different components of the model and the fact that they have the lowest variability in LGA across all subjects, it could be worthwhile to investigate whether the similar answers for LGA is a result of careless responses or simply outstanding consistency in actual LGA.

Sensitivity analyses were conducted by excluding the scale model, i.e., using standard MRMs. The sensitivity analysis results still acknowledge the strong influence of subjects 49 and 69 on the location model but do not reveal significant influence from subjects 7 and 12. These findings are consistent with results in Section “Simulation study”, that is, subjects with influential data in terms of scale can often be neglected if influence analyses are conducted via MRMs only.

PROC NLMIXED in SAS OnDemand for Academics (SAS Institute Inc.) was used to estimate both the original and the leave-one-out MELS models. All the influence analyses using the results from SAS were carried out in R version 4.2.0 (R Core Team). Both the SAS codes and the R codes we used are included in the supporting information.

### Simulation study

**Fig. 3:**
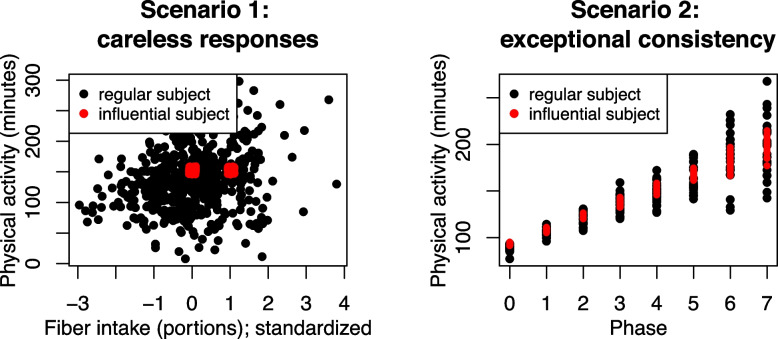
Simulated data examples. Points in black represent data from one regular subject, and points in red represent data from one influential subject

To further illustrate the advantages of conducting influence analyses using MELS models compared to analyses using MRMs with no scale components, we generated simulated examples in two different scenarios, with 500 datasets created for each scenario. Every dataset contains 50 subjects that follow the structure described in Eqs. 1 and 2 before designating some subject(s) to be influential. Both scenarios’ responses resemble daily physical activity time in minutes, so negative values were removed. Examples of one regular subject and one artificially influential subject simulated in each scenario are illustrated in Fig. 3.

The proposed method requires fitting a MELS model, for which estimation can be time-consuming, for every subject in the dataset. Hence, all the MELS models involved in the simulation study were estimated using a fast estimation algorithm for MELS models, FastRegLS, developed by Gill and Hedeker [20]. The supporting information contains codes that utilize FastRegLS to carry out the influence analysis. One or more of the models failed to converge properly for 1 to 18 datasets (mean = 7.67) in the six simulation studies, and these datasets are not included in the result summary. One drawback of the FastRegLS algorithm is that it doesn’t offer log-likelihoods. Hence, the simulation studies primarily concentrate on point estimates, along with the variances and covariances of parameter estimates.

#### Simulation scenario 1

In the first scenario, the time-varying covariate, portions of fiber intake, is continuous and was simulated based on a $\mathcal {N}(3, 1)$ distribution, after which absolute values were taken to ensure non-negativity. The values of the parameters used to generate the simulated datasets are as follows: $\beta = [100, 10]$; $\tau = [7, 0.5]$; $\eta = [20.09, 1, 0.45]$. In other words, the model used to generate data except for the influential one(s) is as follows:

13 $$\begin{aligned} y_{ij}{} & {} = 100 + \nu _i + 10x_{1ij} + \epsilon _{ij}, \nonumber \\ \sigma ^2_{\epsilon _{ij}}{} & {} = \textrm{exp}(7 + \omega _i+0.5x_{1ij}), \\ \left( \begin{array}{c} \nu _i \\ \omega _i \end{array}\right){} & {} \sim \mathcal {N}\left( \left( \begin{array}{c} 0 \\ 0 \end{array}\right) , \left( \begin{array}{cc} 20.09 &{} 0.45 \\ 0.45 &{} 1 \end{array}\right) \right) . \nonumber \end{aligned}$$

There are 500 observations from each subject, and the covariate is standardized for influence analysis to facilitate model estimation. This scenario focuses on the case of careless responses, so we first designated subject 1 to have all responses at 150 and 155 and all covariates at 3 and 4 before standardization. The percentage of simulations in which each influence measure detects subject 1 to be the most influential are summarized in the first column of Table 3. In summary, subject 1 always has the smallest $\text {COVRATIO}^{\tau }$ and the largest $\text {DFBETAS}^{\sigma ^2_{\omega }}$. It also almost always has the largest $\text {DFBETAS}^{\tau _0}$. The second column in Table 3 shows the percentage of detection when we specify the first three subjects to have such careless responses, and the three subjects still almost always have the smallest $\text {COVRATIO}^{\tau }$’s and the largest $\text {DFBETAS}^{\sigma ^2_{\omega }}$’s. Note that only all three influential subjects among the top three are counted as one detection.

#### Simulation scenario 2

For scenario 2, the covariate values were simulated to represent discrete time periods with values that range from 0 to 7, and each subject had 25 observations from each time period before removing negative values. The simulated datasets were generated according to the following structure:

14 $$\begin{aligned} y_{ij}{} & {} = 100 + \nu _i + 15x_{1ij} + \epsilon _{ij}, \nonumber \\ \sigma ^2_{\epsilon _{ij}}{} & {} = \textrm{exp}(3 + \omega _i+0.6x_{1ij}), \\ \left( \begin{array}{c} \nu _i \\ \omega _i \end{array}\right){} & {} \sim \mathcal {N}\left( \left( \begin{array}{c} 0 \\ 0 \end{array}\right) , \left( \begin{array}{cc} 20.09 &{} 0.22 \\ 0.22 &{} 0.25 \end{array}\right) \right) . \nonumber \end{aligned}$$

The influential subject(s) are subject(s) with exceptional consistency throughout the behavioral study and are simulated with an intercept of 0 in the scale model. In the simulation study with the first subject like this, the subject almost always has the largest $C^{\tau }$, the smallest $\text {COVRATIO}^{\tau }$, the largest $\text {DFBETAS}^{\tau _0}$, and the largest $\text {DFBETAS}^{\sigma ^2_{\omega }}$. The same results remain in terms of $\text {COVRATIO}^{\tau }$ and $\text {DFBETAS}^{\sigma ^2_{\omega }}$ when the number of artificial influential subjects increases to three.

As shown in the third column of Table 3, using standard MRMs, none of the influence measures recognize the influences of the designated influential subjects by more than half of the time in either scenario. Therefore, in order to successfully identify an influential subject in these scenarios, it is essential to take the scale model into consideration.

**Table 3:** Percentage of detection using MELS models and MRMs and different influence measures. For scenarios with the first subject as the influential subject, “large(%)" represents the percentage of simulations in which this subject has the largest value of the respective influence measure among all subjects, and “small(%)" represents the percentage of simulations in which this subject has the smallest value of the respective influence measure among all subjects. For scenarios with the first three subjects as the influential subjects, “large(%)" represents the percentage of simulations in which all three of these subjects have the largest values of the respective influence measure among all subjects, and “small(%)" represents the percentage of simulations in which all these three subjects have the smallest values of the respective influence measure among all subjects. Results in the column named “MRMs" were obtained through analyses of using MRMs with no scale components. The simulated datasets used in the MELS model and MRM analyses are the same in each scenario

	MELS models		MRMs
	Single	Multiple (3)	Single
**Scenario 1**			
$C^{\tau }$ [large(%)]	75.9	16.7	-
$\text {COVRATIO}^{\tau }$[large(%)/small(%)]	0/100	0/99.6	-
$\text {DFBETAS}^{\tau _0}$ [large(%)/small(%)]	99.4/0	25.4/0.4	-
$\text {DFBETAS}^{\tau _1}$ [large(%)/small(%)]	5.4/34.6	1.4/15.7	-
$\text {DFBETAS}^{\sigma ^2_{\omega }}$ [large(%)/small(%)]	100/0	99.6/0	-
$\text {C}^{\beta }$ [large(%)]	3.5	5.8	0
$\text {COVRATIO}^{\beta }$[large(%)/small(%)]	15.1/0.8	2.0/0.8	41.6/0
$\text {DFBETAS}^{\beta _0}$ [large(%)/small(%)]	0.8/1.7	0.6/0.6	0/0
$\text {DFBETAS}^{\beta _1}$ [large(%)/small(%)]	21.0/44.4	3.6/22.7	6.8/13.9
$\text {DFBETAS}^{\sigma ^2_{\nu }}$ [large(%)/small(%)]	0.6/0	1.0/0	0/20.1
**Scenario 2**			
$C^{\tau }$ [large(%)]	99.8	62.3	-
$\text {COVRATIO}^{\tau }$[large(%)/small(%)]	0/100	0/99.8	-
$\text {DFBETAS}^{\tau _0}$ [large(%)/small(%)]	99.8/0	69.9/0	-
$\text {DFBETAS}^{\tau _1}$ [large(%)/small(%)]	11.3/16.0	0.4/0.8	-
$\text {DFBETAS}^{\sigma ^2_{\omega }}$ [large(%)/small(%)]	100/0	100/0	-
$\text {C}^{\beta }$ [large(%)]	21.9	0	1.2
$\text {COVRATIO}^{\beta }$ [large(%)/small(%)]	0/2.6	0/0	2.0/1.2
$\text {DFBETAS}^{\beta _0}$ [large(%)/small(%)]	1.4/2.0	0/0	1.4/1.6
$\text {DFBETAS}^{\beta _1}$ [large(%)/small(%)]	38.9/44.9	0/0.8	1.4/0.6
$\text {DFBETAS}^{\sigma ^2_{\nu }}$ [large(%)/small(%)]	1.4/6.3	0/0	1.2/2.0

## Discussion

In this paper, we have discussed procedures to detect subject(s) influential in model fit and parameter estimates in MELS models. This approach allows researchers to identify subjects influential on the scale structure of the outcome being modeled in addition to the location structure. We hope that our method is able to help researchers, especially researchers interested in studying intra-individual variability, better identify interesting or troublesome subjects that they want to study further in an EMA study or a large-scale longitudinal clinical trial. After determining which subjects are considered influential, researchers can keep these subjects in the analysis using our leave-one-out MELS model described in Section “Influence analysis”, and a summary of other common ways of dealing with influential data can be found in a recent work by Aguinis et al. [21]. The proposed method can also benefit analyses not carried out in MELS models by providing researchers with a better understanding of both the location and the scale structures of their data during exploratory analyses.

Our study has focused on subject-level influence analyses, so one possible extension is the detection of influential observations in MELS model. Given that there could be influential observation(s) within a non-influential subject, estimating a separate model for each observation might be required. Because of the enormous number of observations in intensive longitudinal data, such methods can be extremely computationally intensive. This article has also focused on maximum likelihood estimates of 2-level MELS models for normally distributed continuous outcomes. Further development can extend the proposed framework to accommodate models with 3 or more levels [22, 23], models on outcomes with more complicated structures [24], and Bayesian estimation approaches [25]. Future work will also extend to ordinal MELS models [26].

To plan for further data analysis based on the influence analysis results, we recommend readers go through all the results from our framework and use their domain knowledge to decide whether specific subject(s) are considered influential and need further analysis. However, we understand that one might want cutoffs to guide their judgment. Rule-of-thumb cut-off values are $\frac{4}{N}$ for Cook’s distances, $1\pm 3(\frac{r_{\gamma }}{N})$ for COVRATIO, and $\frac{2}{\sqrt{N}}$ for DFBETAS [17]. Also, a possible future direction of this work is to improve existing cut-off values to be more suitable for MELS models.

The simulation studies have showcased that at least some of the influence measures are able to capture all influential subjects in the case that multiple of them coexist. Nevertheless, not all measures perform the same, which might be attributed to the masking effect [27]. Therefore, we again recommend readers carefully examine all influence measures mentioned in the framework, and a future step of this study will be improving individual influence measures to overcome any possible masking effect.

## Conclusion

The proposed influence analysis framework using MELS models enables detection of influential subjects on the scale structure and/or location structure of intensive longitudinal data. Thus, it can facilitate modeling that accounts for the abnormality of certain subject(s). Such benefits of the proposed methods are revealed in both the real-life example and the simulated examples.

### Supplementary Information

**Additional file 1.**

## Data Availability

The dataset used in the health behavior study example are publicly available in Harvard Dataverse, https://dataverse.harvard.edu/dataset.xhtml?persistentId=doi:10.7910/DVN/27388. Code used to generate data in the simulated study is included in the Supporting Information.

## Abbreviations

MELS: Mixed-effects location scale
MRMs: Mixed-effects regression models
BS: Between-subject
WS: Within-subject
EMA: Ecological momentary assessments
FDR: False discovery rate
SQ: Sleep quality
LGA: Learning goal achievement
