# Supplementary material for: Detecting influential subjects in intensive longitudinal data using mixed-effects location scale models
Source: BMC Med Res Methodol. 2023 Oct 18;23:237. doi: 10.1186/s12874-023-02046-9 (PMC10585916; doi:10.1186/s12874-023-02046-9)
Supplement: Supplementary file 1 — Additional file 1. [file 12874_2023_2046_MOESM1_ESM.pdf]

Supplementary Material

This document contains condes for major analyses presented in the paper.

1 Leave-one-out MELS models in SAS

```
* Get the starting values for PROC NLMIXED;

PROC MIXED DATA = health_behav2 METHOD = ml COVTEST;

CLASS id;

MODEL lga = sq/ SOLUTION;

RANDOM intercept/TYPE=un SUBJECT =id;

REPEATED/LOCAL = EXP(sq);

ODS OUTPUT SolutionF = beta;

ODS OUTPUT CovParms = random;

RUN;


* MELS Model in which all subjects are treated equally;

* Results used as starting values for leave-one-out MELS models;

PROC NLMIXED DATA = health_behav2 TECH = quanew GCONV=1e-12 METHOD = gauss

COV ECOV;

PARMS beta0 = 1.8795 beta_sq = 0.1038

tau0 =  -0.2172857 tau_sq = -0.00898

alpha0 = -1.129793

StdDev_scale = 0.05

corr = 0.05;


varBS = EXP(alpha0);
```

```
nu = SQRT(varBS) * theta1;

omega = StdDev_scale * theta2;

mu = beta0  + beta_sq * sq + nu;
varWS = EXP(tau0 + tau_sq * sq + omega);
MODEL lga ~ NORMAL(mu, varWS);
RANDOM theta1 theta2 ~ NORMAL([0,0], [1, corr, 1]) SUBJECT = id;

* Get the estimates for variances and covariance of random effects;
ESTIMATE "varBS" EXP(alpha0);
ESTIMATE "varWS" StdDev_scale**2;
ESTIMATE "COV" corr * StdDev_scale * SQRT(EXP(alpha0));

ODS OUTPUT ParameterEstimates = healthbehav_all_parm;
ODS OUTPUT FitStatistics= healthbehav_all_fit;
ODS OUTPUT CovMatParmEst= healthbehav_all_cov;
ODS OUTPUT AdditionalEstimates= healthbehav_all_est;
ODS OUTPUT CovMatAddEst= healthbehav_all_est_cov;

RUN;

* Get all the distinct IDs in the data set;
PROC SQL NOPRINT;
  SELECT DISTINCT id INTO :unique_id separated by ' ' FROM health_behav2;
QUIT;
```

```
* Re-estimate the model with each subject separated from level 2 of
* both the location part and the scale part of the model
* Create empty data sets to store the results;
DATA outlier.sub_parm;
FORMAT ID 6.
Parameter $12.
Estimate D8.4
StandardError D8.4
DF BEST4.
tValue 7.2
Probt PVALUE6.4
Alpha BEST5.
Lower D8.5
Upper D8.4
Gradient D8.5;
STOP;
RUN;

DATA outlier.sub_est;
FORMAT ID 6.
Label $5.
Estimate D8.4
StandardError D8.4
DF BEST4.
tValue 7.2
Probt PVALUE6.4
Alpha BEST5.
Lower D8.5
```

```
Upper D8.4;
STOP;
RUN;

DATA outlier.sub_fit;
FORMAT ID 6.
Descr $40.
Value D6.1;
STOP;
RUN;

DATA outlier.sub_cov;
FORMAT ID 6.
Parameter $12.
StdDev_scale D8.4
alpha0 D8.4
beta0 D8.4
beta_sq D8.4
c D8.4
c_sq D8.4
corr D8.4
d D8.4
d_sq D8.4
tau0 D8.4
tau_sq D8.4;
STOP;
RUN;
```

```
DATA outlier.sub_est_cov;
FORMAT ID 6.
Label $5.
Cov1 D8.4
Cov2 D8.4
Cov3 D8.4;
STOP;
RUN;*/

* Re-estimate the model with each subject SEPARATED from the estimation of ;
* both random location effects and random scale effects;
%MACRO INFLUENTIAL;
/*store:
1. sub_parm: fixed effect estimates
2. sub_fit: model fit statistics (-2 log likelihood, AIC, AICC, BIC)
3. sub_cov: covariance matrix of fixed effect estimates
4. sub_est: estimates of variances and covariance of random effects
5. sub_est_cov: variance-covariance matrix of the variances and
covariance of random effects*/
%DO i = 1 %TO %SYSFUNC(COUNTW(&unique_id));
%LET this_id = %SCAN(&unique_id,&i,%STR( ));
ODS OUTPUT ParameterEstimates = outlier.sub_parm_new;
ODS OUTPUT FitStatistics = outlier.sub_fit_new;
ODS OUTPUT CovMatParmEst = outlier.sub_cov_new;
ODS OUTPUT AdditionalEstimates= outlier.sub_est_new;
ODS OUTPUT CovMatAddEst= outlier.sub_est_cov_new;
```

```
PROC NLMIXED DATA = r2_sim.health_behav2 TECH = quanew GCONV=1e-12
 METHOD = gauss COV ECOV;
PARMS beta0 = 1.8795 beta_sq = 0.1038
tau0 =  -0.2172857 tau_sq = -0.00898
alpha0 = -1.129793
StdDev_scale = 0.0001
c = 0.0001
d = 0.0001
corr = 0.0001;

varBS = EXP(alpha0);
nu = SQRT(varBS) * theta1;

omega = StdDev_scale * theta2;

mu = beta0 * (id ~= &this_id) + beta_sq * sq + nu * (id ~= &this_id) +
 c * (id = &this_id);
varWS = EXP(tau0 * (id ~= &this_id) + tau_sq * sq + omega * (id ~= &this_id) +
 d * (id = &this_id));
MODEL lga ~ NORMAL(mu, varWS);
RANDOM theta1 theta2 ~ NORMAL([0,0], [1, corr, 1]) SUBJECT = id;
ESTIMATE "varBS" EXP(alpha0);
ESTIMATE "varWS" StdDev_scale**2;
ESTIMATE "COV" corr * StdDev_scale * SQRT(EXP(alpha0));

RUN;
```

```
* Select variables and add subject ID;
DATA outlier.sub_parm_new;
       SET outlier.sub_parm_new;
       id=&this_id;
RUN;

DATA outlier.sub_est_new;
       SET outlier.sub_est_new;
       id=&this_id;
RUN;

DATA outlier.sub_fit_new;
       SET outlier.sub_fit_new;
       id=&this_id;
       KEEP Descr Value ID;
RUN;

DATA outlier.sub_cov_new;
       SET outlier.sub_cov_new;
       id=&this_id;
       DROP Row;
RUN;

DATA outlier.sub_est_cov_new;
       SET outlier.sub_est_cov_new;
       id=&this_id;
       DROP Row;
```

```
RUN;

    PROC APPEND BASE=outlier.sub_parm NEW=outlier.sub_parm_new;
    RUN;

    PROC APPEND BASE=outlier.sub_est NEW=outlier.sub_est_new;
    RUN;

    PROC APPEND BASE=outlier.sub_fit NEW=outlier.sub_fit_new;
    RUN;

    PROC APPEND BASE=outlier.sub_cov NEW=outlier.sub_cov_new;
    RUN;

    PROC APPEND BASE=outlier.sub_est_cov NEW=outlier.sub_est_cov_new;
    RUN;

%END;
%MEND INFLUENTIAL;

%INFLUENTIAL;
```

2 Influence analysis in R

```
library(tidyverse)

# Influence on model fit
```

```
# Format fit data from long to wide
all_fit2 <- all_fit %>%
  spread(key = "Descr", value = "Value")
all_fit3 <- as.vector(as.matrix(all_fit2))
sub_fit2 <- sub_fit %>%
  spread(key = "Descr", value = "Value")
sub_fit3 <- as.matrix(sub_fit2)

fit_compare <- as.data.frame(cbind("ID" = sub_fit2[,"ID"],
-t(t(sub_fit3[,2:5]) - all_fit3)))
colnames(fit_compare)[1] <- "ID"
fit_compare %>%
  arrange(desc(`-2 Log Likelihood`))

# Likelihood ratio tests with FDR controlling procedures
p <- cbind(fit_compare$ID, 1-pchisq(fit_compare$`-2 Log Likelihood`, df = 2))
p[order(p.adjust(p[,2], method = "fdr")), ]



# Cook's distances
# Creates an empty data set to store the results
cook_dist <- data.frame("fixed_location" = numeric(n_sub),
    "fixed_scale" = numeric(n_sub),
    "random" = numeric(n_sub))

# Fixed location effects
for (i in 1:n_sub){
```

```
  sub_cov_temp <- sub_cov2[((i-1) * 9 + 1):(9*i),]
  rownames(sub_cov_temp) <- cov_names
  # select variables to be used to calculate Cook's distance
  select <- c("beta0", "beta_sq")
  sub_cov_select <- sub_cov_temp[select, select]
  sub_parm_temp <- sub_parm[((i-1) * 9 + 1):(9*i),]
  sub_parm_select <- sub_parm_temp[sub_parm_temp$Parameter %in% select, "Estimate"]
  all_parm_select <- all_parm[all_parm$Parameter %in% select, "Estimate"]
  cook_dist[i, "fixed_location"] = (1/2) * t(all_parm_select - sub_parm_select) %*%
    solve(as.matrix(sub_cov_select)) %*% (all_parm_select - sub_parm_select)
}

# Fixed scale effects
for (i in 1:n_sub){
  sub_cov_temp <- sub_cov2[((i-1) * 9 + 1):(9*i),]
  rownames(sub_cov_temp) <- cov_names
  # select variables to be used to calculate Cook's distance
  select <- c("tau0", "tau_sq")
  sub_cov_select <- sub_cov_temp[select, select]
  sub_parm_temp <- sub_parm[((i-1) * 9 + 1):(9*i),]
  sub_parm_select <- sub_parm_temp[sub_parm_temp$Parameter %in% select, "Estimate"]
  all_parm_select <- all_parm[all_parm$Parameter %in% select, "Estimate"]
  cook_dist[i, "fixed_scale"] = (1/2) * t(all_parm_select - sub_parm_select) %*%
    solve(as.matrix(sub_cov_select)) %*% (all_parm_select - sub_parm_select)
}

# Random effects
```

```
for (i in 1:n_sub){
  sub_cov_temp <- sub_est_cov2[((i-1) * 3 + 1):(3*i),]
  sub_parm_temp <- sub_est[((i-1) * 3 + 1):(3*i),"Estimate"]
  all_parm_temp <- all_est[, "Estimate"]
  cook_dist[i, "random"] = (1/3) * t(all_parm_temp - sub_parm_temp) %*%
    solve(as.matrix(sub_cov_temp)) %*% (all_parm_temp - sub_parm_temp)
}

cook_dist <- cbind("ID" = sub_fit2$ID, cook_dist)



# DFBETAS
# Creates an empty data set to store the results
dfbetas <- matrix(numeric(n_sub * 7), nrow = n_sub)
rnames <- c("beta0", "beta_sq", "tau0", "tau_sq", "varBS", "varWS", "COV")
colnames(dfbetas) <- rnames

for (i in 1:n_sub){
    sub_parm_temp1 <- sub_parm[((i-1) * 9 + 1):(9*i),]
    sub_parm_temp2 <- sub_est[((i-1) * 3 + 1):(3*i),]
    for (j in 1:4){
      dfbetas[i,j] <- (all_parm$Estimate[all_parm$Parameter == rnames[j]] -
        sub_parm_temp1$Estimate[sub_parm_temp1$Parameter == rnames[j]])/
      sub_parm_temp1$StandardError[sub_parm_temp1$Parameter == rnames[j]]
    }
    dfbetas[i, 5:7] <- (all_est$Estimate - sub_parm_temp2$Estimate)/
    sub_parm_temp2$StandardError
```

```
}

dfbetas <- as.data.frame(cbind("ID" = sub_fit2$ID, dfbetas))


# COVRATIO
# Creates an empty data set to store the results
COVRATIO_result <- data.frame("fixed_location" = numeric(n_sub),
    "fixed_scale" = numeric(n_sub),
    "random" = numeric(n_sub))

# Fixed location effects
for (i in 1:n_sub){
  sub_cov_temp <- sub_cov2[((i-1) * 9 + 1):(9*i),]
  rownames(sub_cov_temp) <- cov_names
  # select variables to be used to calculate COVRATIO
  select <- c("beta0", "beta_sq")
  sub_cov_select <- sub_cov_temp[select, select]
  all_cov_select <- all_cov[all_cov$Parameter %in% select,
  colnames(all_cov) %in% select]
  COVRATIO_result[i, "fixed_location"] = det(as.matrix(sub_cov_select))/
  det(as.matrix(all_cov_select))
}

# Fixed scale effects
for (i in 1:n_sub){
  sub_cov_temp <- sub_cov2[((i-1) * 9 + 1):(9*i),]
```

```
  rownames(sub_cov_temp) <- cov_names
  # select variables to be used to calculate COVRATIO
  select <- c("tau0", "tau_sq")
  sub_cov_select <- sub_cov_temp[select, select]
  all_cov_select <- all_cov[all_cov$Parameter %in% select,
  colnames(all_cov) %in% select]
  COVRATIO_result[i, "fixed_scale"] = det(as.matrix(sub_cov_select))/
  det(as.matrix(all_cov_select))
}

# Random effects
for (i in 1:n_sub){
  sub_cov_temp <- sub_est_cov2[((i-1) * 3 + 1):(3*i),]
  COVRATIO_result[i, "random"] = det(as.matrix(sub_cov_temp))/
  det(as.matrix(all_est_cov[, -(1:2)]))
}
```

3 Generation of simulated data in R

```
# Scenario 1: careless responses (1 influential subject)
set.seed(11)

yij <- list()
X <- list()
subject <- list()
```

```
# Generating parameters
beta <- c(100, 10)
alpha0 <- 3
tau <- c(7, 0.5)
ssigma_omega <- 1
rho_v_omega <- 0.1
sigma_v_omega <- matrix(c(1, rho_v_omega, rho_v_omega, 1), nrow = 2)

for (i in 1:500){
  X[[i]] <- cbind(1, abs(rnorm(n=50*500, mean=3, sd = 1)))
  theta_i <- mvrnorm(n = 50, mu = c(0,0), Sigma = sigma_v_omega)
  theta1_ij <- rep(theta_i[,1], each = 500)
  theta2_ij <- rep(theta_i[,2], each = 500)
  psi_ij <- rnorm(n=50*500, mean = 0, sd = 1)
  vij <- sqrt(exp(alpha0)) * theta1_ij
  eij <- sqrt(exp(X[[i]] %*% tau + sqrt(ssigma_omega) * theta2_ij)) * psi_ij
  yij[[i]] <- X[[i]] %*% beta + vij + eij

  X[[i]][1:500,2] <- sample(c(3,4), 500, replace = TRUE)
  yij[[i]][1:500] <- sample(c(150, 155), 500, replace = TRUE)
  y_select <- yij[[i]] >= 0
  X[[i]] <- X[[i]][y_select, ]
  X[[i]][, 2] <- (X[[i]][, 2] - mean(X[[i]][, 2]))/sd(X[[i]][, 2]) # Standardize X
  subject[[i]] <- rep(1:50, each = 500)[y_select]
  yij[[i]] <- yij[[i]][y_select]
}
```

```
# Scenario 1: careless responses (3 influential subjects)
set.seed(21)
yij <- list()
X <- list()
subject <- list()

# Generating parameters
beta <- c(100, 10)
alpha0 <- 3
tau <- c(7, 0.5)
ssigma_omega <- 1
rho_v_omega <- 0.1
sigma_v_omega <- matrix(c(1, rho_v_omega, rho_v_omega, 1), nrow = 2)

for (i in 1:500){
  X[[i]] <- cbind(1, abs(rnorm(n=50*500, mean=3, sd = 1)))
  theta_i <- mvrnorm(n = 50, mu = c(0,0), Sigma = sigma_v_omega)
  theta1_ij <- rep(theta_i[,1], each = 500)
  theta2_ij <- rep(theta_i[,2], each = 500)
  psi_ij <- rnorm(n=50*500, mean = 0, sd = 1)
  vij <- sqrt(exp(alpha0)) * theta1_ij
  eij <- sqrt(exp(X[[i]] %*% tau + sqrt(ssigma_omega) * theta2_ij)) * psi_ij
  yij[[i]] <- X[[i]] %*% beta + vij + eij
  X[[i]][1:1500,2] <- sample(c(3,4), 1500, replace = TRUE)
  yij[[i]][1:1500] <- sample(c(150, 155), 1500, replace = TRUE)

  y_select <- yij[[i]] >= 0
```

```
  X[[i]] <- X[[i]][y_select, ]
  X[[i]][, 2] <- (X[[i]][, 2] - mean(X[[i]][, 2]))/sd(X[[i]][, 2]) # Standardize X
  subject[[i]] <- rep(1:50, each = 500)[y_select ]
  yij[[i]] <- yij[[i]][y_select]
}

# Scenario 2: exceptional consistency (1 influential subject)
set.seed(15)
yij <- list()
X <- list()
subject <- list()

# Generating parameters
beta <- c(100, 15)
alpha0 <- 3
tau <- c(3, 0.6)
ssigma_omega <- 0.25
rho_v_omega <- 0.1
sigma_v_omega <- matrix(c(1, rho_v_omega, rho_v_omega, 1), nrow = 2)

for (i in 1:500){
  X[[i]] <- cbind(1, rep(rep(0:7, each = 25), 50))
  theta_i <- mvrnorm(n = 50, mu = c(0,0), Sigma = sigma_v_omega)
  theta1_ij <- rep(theta_i[,1], time = rep(200, 50))
  theta2_ij <- rep(theta_i[,2], time = rep(200, 50))
  psi_ij <- rnorm(n = 200 * 50 , mean = 0, sd = 1)
  vij <- sqrt(exp(alpha0)) * theta1_ij
```

```
  eij <- sqrt(exp(X[[i]][,1:2] %*% tau + sqrt(ssigma_omega) * theta2_ij)) *
  psi_ij
  yij_temp2 <- X[[i]][201:(200 * 50),] %*% beta + vij[201:(200 * 50)] +
  eij[201:(200 * 50)]
  yij_temp1 <- X[[i]][1:200,] %*% beta + vij[1:200] +
  sqrt(exp(X[[i]][1:200,1:2] %*% tau - 3)) *
  psi_ij[1:200]
  yij[[i]] <- c(yij_temp1, yij_temp2)

  X[[i]] <- X[[i]][yij[[i]] >= 0, ]
  subject[[i]] <- rep(1:50, time =  rep(200, 50))[yij[[i]] >= 0]
  yij[[i]] <- yij[[i]][yij[[i]] >= 0]
}

# Scenario 2: exceptional consistency (3 influential subjects)
set.seed(35)
yij <- list()
X <- list()
subject <- list()

# Generating parameters
beta <- c(100, 15)
alpha0 <- 3
tau <- c(3, 0.6)
ssigma_omega <- 0.25
rho_v_omega <- 0.1
sigma_v_omega <- matrix(c(1, rho_v_omega, rho_v_omega, 1), nrow = 2)
```

```
for (i in 1:500){
  X[[i]] <- cbind(1, rep(rep(0:7, each = 25), 50))
  theta_i <- mvrnorm(n = 50, mu = c(0,0), Sigma = sigma_v_omega)
  theta1_ij <- rep(theta_i[,1], time = rep(200, 50))
  theta2_ij <- rep(theta_i[,2], time = rep(200, 50))
  psi_ij <- rnorm(n = 200 * 50 , mean = 0, sd = 1)
  vij <- sqrt(exp(alpha0)) * theta1_ij
  eij <- sqrt(exp(X[[i]][,1:2] %*% tau + sqrt(ssigma_omega) * theta2_ij)) *
  psi_ij
  yij_temp2 <- X[[i]][601:(200 * 50), ] %*% beta + vij[601:(200 * 50)] +
  eij[601:(200 * 50)]
  yij_temp1 <- X[[i]][1:600,] %*% beta + vij[1:600] +
  sqrt(exp(X[[i]][1:600,1:2] %*% tau - 3)) *
  psi_ij[1:600]
  yij[[i]] <- c(yij_temp1, yij_temp2)

  X[[i]] <- X[[i]][yij[[i]] >= 0, ]
  subject[[i]] <- rep(1:50, time =  rep(200, 50))[yij[[i]] >= 0]
  yij[[i]] <- yij[[i]][yij[[i]] >= 0]
}
```

4 Influence analysis using the FastRegLS algorithm

The functions `fastregls` and `fastregls_sub` attached are slightly modified versions of the original FastRegLS algorithm proposed by Gill and Hedeker [1]. Specifically, the following `fastregls` function additionally returns the variance-covariance matrices of parameter estimates, and the `fastregls_sub` function fits a model in which the

subject specified by the `subject` argument is separated from the estimation of the fixed and random intercepts. Note that this example codes can only accommodate models with one time varying covariate and random intercepts but no random slopes in both the location and the scale models. Additional adjustments are needed for more complicated MELS models.

```
library(lme4)
library(numDeriv)
source("auxFunctions.R")
library(MASS)
options(warn = 1)


fastregls = function(y, X, Z, id, nIter, getSigmaSE = 0) {
  k = dim(X)[2]
  #m = dim(U)[2]
  p = dim(Z)[2]
  id.factor = as.factor(id)
  #Create a vector with the group sizes
  groupSizes = tabulate(id, nbins = max(id))
  #create a vector with indices of first observation of each subject
  inds = rep(0, max(id))
  inds[1] = 1
  for (i in 2:length(inds)) {
    inds[i] = inds[i-1] + groupSizes[i-1]
  }
  mod = lmer(y~X-1+(1|id.factor), REML = FALSE)
  #Initialize beta
  betaHat = unname(summary(mod)$coef[,1])
```

```
blups = repVec(ranef(mod)$id$'(Intercept)', groupSizes)
delta = y-X%*%betaHat-blups
tempResponse = log(delta^2)
#standardizedBlups =
#blups/(sqrt(summary(mod)$varcor$id[[1]]))
mod = lmer(tempResponse~Z-1+ blups + (1|id.factor), REML = FALSE)
#Initialize alpha
alphaHat = unname(summary(mod)$coef[,1])[1:p] + c(1.2704, rep(0, p-1))
#Initialize rho
corrHat = unname(summary(mod)$coef[,1])[p+1]
gammaHat = repVec(ranef(mod)$id$'(Intercept)', groupSizes)
#tempResponse = log(blups[inds]^2)
#Initialize tau
#tauHat = unname(lm(tempResponse~U[inds,]-1)$coef) + c(1.2704, rep(0, m-1))
sigma_sq_nu_hat <- summary(mod)$varcor$id[[1]]
#set sigmaSE to 0
sigmaSE = 0
for (h in 1:nIter) {
  #Estimate random effects
  a = (y-X%*%betaHat)/exp(Z %*% alphaHat + corrHat*blups + gammaHat)
  a = sumVec(a, groupSizes)
  b = ( 1/sigma_sq_nu_hat + sumVec(1/exp(Z %*% alphaHat +
                                          corrHat*blups + gammaHat), groupSizes))^(-1)
  blups = as.vector(a*b)
  blups = repVec(blups, groupSizes)
  #standardizedBlups = blups/repVec(exp(t(as.matrix(0.5*U[inds,]) %*%
  #tauHat)), groupSizes)
```

```
#if (h <= nIter) {
#Estimate beta
tempResponse = y - blups
#mod = lmer(y~X-1 + (1|id.factor),
#weights = exp(-Z%*%alphaHat- corrHat*blups-gammaHat))
mod = lm(tempResponse~X-1,
         weights = exp(-Z%*%alphaHat- corrHat*blups-gammaHat))
betaHat = unname(summary(mod)$coef[,1])
betaSE = diag(vcov(mod))^0.5
#} else {
#mod = lmer(y~X-1 + (1|id.factor), REML = FALSE,
#weights = exp(-Z%*%alphaHat- corrHat*blups-gammaHat))
#betaSE = diag(vcov(mod))^0.5
#betaHat = unname(summary(mod)$coef[,1])
#}
#tempResponse = y - blups
tempResponse = log((y - blups - X %*% betaHat)^2)
#standardizedBlups = blups/repVec(exp(t(as.matrix(U[inds,]) %*%
#tauHat)), groupSizes)
mod = lmer(tempResponse~Z-1+ blups +(1|id.factor), REML = FALSE)
#Estimate alpha
alphaHat = unname(summary(mod)$coef[,1])[1:p] + c(1.2704, rep(0, p-1))
alphaSE = diag(vcov(mod))[1:p]^0.5
alpha_vcov = vcov(mod)[1:p, 1:p]
#Estimate rho
corrHat = unname(summary(mod)$coef[,1])[p+1]
corrSE = diag(vcov(mod))[p+1]^0.5
```

```
  gammaHat = repVec(ranef(mod)$id$'(Intercept)', groupSizes)
  #Estimate random scale variance
  sigmaSqHat = summary(mod)$varcor$id[[1]]
  #estimate random scale variance se
  if (getSigmaSE == 1 && h == nIter) {
    #model = mod
    dd.ML <- lme4:::devfun2(mod,useSc=TRUE,signames=FALSE)
    vv <- as.data.frame(VarCorr(mod))
    pars <- vv[,"sdcor"]
    hh1 <- numDeriv::hessian(dd.ML,pars)
    vv2 <- 2*solve(hh1)
    sigmaSE = sqrt(diag(vv2))[1]
  }
  #Estimate tau
  tempResponse = log(blups[inds]^2)
  #mod = lm(tempResponse~U[inds,]-1)# + offset(offset),
  #weights = 1/blupVariance)
  #tauHat = unname(mod$coef) + c(1.2704, rep(0, m-1))
  #tauSE = diag(vcov(mod))^0.5
  sigma_sq_nu_hat <- exp(mean(tempResponse) + 1.2704)
}
mod = lmer(y~X-1 + (1|id.factor), REML = FALSE,
           weights = exp(-Z%*%alphaHat- corrHat*blups-gammaHat))
betaSE = diag(vcov(mod))^0.5
beta_vcov = vcov(mod)
betaHat = unname(summary(mod)$coef[,1])
#get sigma_squared_nu info
```

```
#sigma_sq_nu_hat <- unname(unlist(summary(mod)$varcor))
sigma_sq_nu_hat_SE <- 0
if (getSigmaSE == 1) {
  sigma_sq_nu_hat <- summary(mod)$varcor$id[[1]]
  dd.ML <- lme4:::devfun2(mod,useSc=TRUE,signames=FALSE)
  vv <- as.data.frame(VarCorr(mod))
  pars <- vv[,"sdcor"]
  hh1 <- numDeriv::hessian(dd.ML,pars)
  vv2 <- 2*solve(hh1)
  sigma_sq_nu_hat_SE = sqrt(diag(vv2))[1]
}
marginal_variances = exp(Z%*%alphaHat + corrHat*blups + gammaHat)
return(list(beta = betaHat,
            sigma_sq_nu = sigma_sq_nu_hat,
            alpha = alphaHat,
            corr = corrHat,
            sigma_sq_omega = sigmaSqHat,
            betaSE = betaSE,
            beta_vcov = beta_vcov,
            sigma_sq_nu_SE = sigma_sq_nu_hat_SE,
            alphaSE = alphaSE,
            alpha_vcov = alpha_vcov,
            corrSE = corrSE,
            sigma_sq_omega_SE = sigmaSE,
            nu = blups,
            omega = gammaHat,
            marginal_variances = marginal_variances
```

```
  ))
}


fastregls_sub = function(y, X, Z, id, nIter, subject, getSigmaSE = 0) {
  k = dim(X)[2] + 2
  #m = dim(U)[2]
  p = dim(Z)[2] + 2
  id.factor = as.factor(id)
  #Create a vector with the group sizes
  groupSizes = tabulate(id, nbins = max(id))
  #create a vector with indices of first observation of each subject
  inds = rep(0, max(id))
  inds[1] = 1
  for (i in 2:length(inds)) {
    inds[i] = inds[i-1] + groupSizes[i-1]
  }
  X = cbind(X, 'intercept.alt' = ifelse(id == subject, 1, 0),
            'intercept.alt2' = ifelse(id == subject, 0, 1))
  mod = lmer(y ~ X-1 + (0 + X[,'intercept.alt2']|id),
             REML = FALSE)
  #Initialize beta
  betaHat = unname(summary(mod)$coef[,1])
  Z = cbind(Z, 'intercept.alt' = ifelse(id == subject, 1, 0),
            'intercept.alt2' = ifelse(id == subject, 0, 1))
  blups = repVec(ranef(mod)$id$'X[, "intercept.alt2"]', groupSizes)
```

```
delta = y-X%*%betaHat-blups
tempResponse = log(delta^2)
#standardizedBlups = blups/(sqrt(summary(mod)$varcor$id[[1]]))
mod = lmer(tempResponse~Z-1+ blups + (0 + Z[, 'intercept.alt2']|id),
           REML = FALSE)
#Initialize alpha
alphaHat = unname(summary(mod)$coef[,1])[1:p] + c(1.2704, rep(0, p-1))
#Initialize rho
corrHat = unname(summary(mod)$coef[,1])[p+1]
gammaHat = repVec(ranef(mod)$id$'Z[, "intercept.alt2"]', groupSizes)
#tempResponse = log(blups[inds]^2)
#Initialize tau
#tauHat = unname(lm(tempResponse~U[inds,]-1)$coef) + c(1.2704, rep(0, m-1))
sigma_sq_nu_hat <- summary(mod)$varcor$id[[1]]
#set sigmaSE to 0
sigmaSE = 0
for (h in 1:nIter) {
  #Estimate random effects
  a = (y-X%*%betaHat)/exp(Z %*% alphaHat + corrHat*blups + gammaHat)
  a = sumVec(a, groupSizes)
  b = ( 1/sigma_sq_nu_hat + sumVec(1/exp(Z %*% alphaHat +
                                          corrHat*blups + gammaHat),
                                   groupSizes))^(-1)
  blups = as.vector(a*b)
  blups = repVec(blups, groupSizes)
  #standardizedBlups = blups/repVec(exp(t(as.matrix(0.5*U[inds,]) %*%tauHat)), groupSizes)
  #if (h <= nIter) {
  #Estimate beta
```

```
tempResponse = y - blups
#mod = lmer(y~X-1 + (1|id.factor),
#weights = exp(-Z%*%alphaHat- corrHat*blups-gammaHat))
mod = lm(tempResponse~X-1,
         weights = exp(-Z%*%alphaHat- corrHat*blups-gammaHat))
betaHat = unname(summary(mod)$coef[,1])
betaSE = diag(vcov(mod))^0.5
#} else {
#mod = lmer(y~X-1 + (1|id.factor), REML = FALSE,
#weights = exp(-Z%*%alphaHat- corrHat*blups-gammaHat))
#betaSE = diag(vcov(mod))^0.5
#betaHat = unname(summary(mod)$coef[,1])
#}
#tempResponse = y - blups
tempResponse = log((y - blups - X %*% betaHat)^2)
#standardizedBlups = blups/repVec(exp(t(as.matrix(U[inds,]) %*%
#tauHat)), groupSizes)
mod = lmer(tempResponse~Z-1+ blups +(0 +
                                       Z[,'intercept.alt2']|id), REML = FALSE)
#Estimate alpha
alphaHat = unname(summary(mod)$coef[,1])[1:p] + c(1.2704, rep(0, p-1))
alphaSE = diag(vcov(mod))[1:p]^0.5
alpha_vcov = vcov(mod)[1:p, 1:p]
#Estimate rho
corrHat = unname(summary(mod)$coef[,1])[p+1]
corrSE = diag(vcov(mod))[p+1]^0.5
gammaHat = repVec(ranef(mod)$id$'Z[, "intercept.alt2"]', groupSizes)
#Estimate random scale variance
```

```
  sigmaSqHat = summary(mod)$varcor$id[[1]]
  #estimate random scale variance se
  if (getSigmaSE == 1 && h == nIter) {
    #model = mod
    dd.ML <- lme4:::devfun2(mod,useSc=TRUE,signames=FALSE)
    vv <- as.data.frame(VarCorr(mod))
    pars <- vv[,"sdcor"]
    hh1 <- numDeriv::hessian(dd.ML,pars)
    vv2 <- 2*solve(hh1)
    sigmaSE = sqrt(diag(vv2))[1]
  }
  #Estimate tau
  tempResponse = log(blups[inds]^2)
  #mod = lm(tempResponse~U[inds,]-1)# + offset(offset),
  #weights = 1/blupVariance)
  #tauHat = unname(mod$coef) + c(1.2704, rep(0, m-1))
  #tauSE = diag(vcov(mod))^0.5
  sigma_sq_nu_hat <- exp(mean(tempResponse) + 1.2704)
}
mod = lmer(y~X-1 +(0 + X[,'intercept.alt2']|id), REML = FALSE,
           weights = exp(-Z%*%alphaHat- corrHat*blups-gammaHat))
betaSE = diag(vcov(mod))^0.5
beta_vcov = vcov(mod)
betaHat = unname(summary(mod)$coef[,1])
#get sigma_squared_nu info
#sigma_sq_nu_hat <- unname(unlist(summary(mod)$varcor))
sigma_sq_nu_hat_SE <- 0
```

```
  if (getSigmaSE == 1) {
    sigma_sq_nu_hat <- summary(mod)$varcor$id[[1]]
    dd.ML <- lme4:::devfun2(mod,useSc=TRUE,signames=FALSE)
    vv <- as.data.frame(VarCorr(mod))
    pars <- vv[,"sdcor"]
    hh1 <- numDeriv::hessian(dd.ML,pars)
    vv2 <- 2*solve(hh1)
    sigma_sq_nu_hat_SE = sqrt(diag(vv2))[1]
  }
  marginal_variances = exp(Z%*%alphaHat + corrHat*blups + gammaHat)
  return(list(beta = betaHat,
              sigma_sq_nu = sigma_sq_nu_hat,
              alpha = alphaHat,
              corr = corrHat,
              sigma_sq_omega = sigmaSqHat,
              betaSE = betaSE,
              beta_vcov = beta_vcov,
              sigma_sq_nu_SE = sigma_sq_nu_hat_SE,
              alphaSE = alphaSE,
              alpha_vcov = alpha_vcov,
              corrSE = corrSE,
              sigma_sq_omega_SE = sigmaSE,
              nu = blups,
              omega = gammaHat,
              marginal_variances = marginal_variances))
}
```

```
result_all <- list()
# Separate each subject from random intercept estimation
for (i in 1:ndat){
  message(paste0("Dataset: ", i))
  tryCatch({y_mod <- as.vector(yij[[i]])
  X_mod <- X[[i]]
  Z_mod <- X[[i]][, 1:2]
  id_mod <- subject[[i]]
  result_all_temp <- fastregls(y = y_mod, X= X_mod, Z = Z_mod,
                              id = id_mod, nIter = 5,
                              getSigmaSE = 1)
  result_all[[i]] <- result_all_temp
  result_subj[[i]] <- list()
  for(k in 1:50) {
    X_mod_subj <- X_mod[,2]
    Z_mod_subj <- Z_mod[,2]
    result_subj_temp <- fastregls_sub(y = y_mod,
                                     as.matrix(X_mod_subj), Z = as.matrix(Z_mod_subj),
                                     id = id_mod, nIter = 5, subject = k, getSigmaSE = 1)
    result_subj[[i]][[k]] <- result_subj_temp
  }},
  error = function(e){
    warnings(paste("An error occurred for dataset", i,":\n"), e)
  })
}

FastRegLS_influence <- function(result_all = NULL, result_subj = NULL, ndat = NULL,
```

```
                              nsubj = 0){
beta_cook <- matrix(NA, nrow = ndat, ncol = nsubj)
beta_covratio <- matrix(NA, nrow = ndat, ncol = nsubj)
tau_cook <- matrix(NA, nrow = ndat, ncol = nsubj)
tau_covratio <- matrix(NA, nrow = ndat, ncol = nsubj)
dfbeta_beta0 <- matrix(NA, nrow = ndat, ncol = nsubj)
dfbeta_beta1 <- matrix(NA, nrow = ndat, ncol = nsubj)
dfbeta_tau0 <- matrix(NA, nrow = ndat, ncol = nsubj)
dfbeta_tau1 <- matrix(NA, nrow = ndat, ncol = nsubj)
dfbeta_omega <- matrix(NA, nrow = ndat, ncol = nsubj)
dfbeta_nu <- matrix(NA, nrow = ndat, ncol = nsubj)
beta1_all <- rep(NA, ndat)
beta1_subj <- matrix(NA, nrow = ndat, ncol = nsubj)
beta1_se_all <- matrix(NA, nrow = ndat, ncol = nsubj)
beta1_se_subj <- matrix(NA, nrow = ndat, ncol = nsubj)
beta0_all <- rep(NA, ndat)
beta0_subj <- matrix(NA, nrow = ndat, ncol = nsubj)
beta0_se_all <- matrix(NA, nrow = ndat, ncol = nsubj)
beta0_se_subj <- matrix(NA, nrow = ndat, ncol = nsubj)
beta_vcov_all <- list()
beta_vcov_subj <- list()
tau1_all <- rep(NA, ndat)
tau1_subj <- matrix(NA, nrow = ndat, ncol = nsubj)
tau1_se_subj <- matrix(NA, nrow = ndat, ncol = nsubj)
tau0_all <- rep(NA, ndat)
tau0_subj <- matrix(NA, nrow = ndat, ncol = nsubj)
tau0_se_subj <- matrix(NA, nrow = ndat, ncol = nsubj)
```

```
tau_vcov_all <- list()
tau_vcov_subj <- list()
sigma_nu_all <-rep(NA, ndat)
sigma_nu_subj <- matrix(NA, nrow = ndat, ncol = nsubj)
sigma_nu_se_subj <- matrix(NA, nrow = ndat, ncol = nsubj)
sigma_omega_all <-rep(NA, ndat)
sigma_omega_subj <- matrix(NA, nrow = ndat, ncol = nsubj)
sigma_omega_se_subj <- matrix(NA, nrow = ndat, ncol = nsubj)
for (i in 1:ndat){
  beta0_all[i] <- result_all[[i]]$beta[1]
  tau0_all[i] <- result_all[[i]]$alpha[1]
  beta1_all[i] <- result_all[[i]]$beta[2]
  tau1_all[i] <- result_all[[i]]$alpha[2]
  sigma_nu_all[i] <- result_all[[i]]$sigma_sq_nu
  sigma_omega_all[i] <- result_all[[i]]$sigma_sq_omega
  beta_vcov_all[[i]] <- result_all[[i]]$beta_vcov[1:2, 1:2]
  tau_vcov_all[[i]] <- result_all[[i]]$alpha_vcov[1:2, 1:2]
  beta_vcov_subj[[i]] <- list()
  tau_vcov_subj[[i]] <- list()
  for(j in 1:nsubj){
    tryCatch({
      beta_vcov_subj[[i]][[j]] <- result_subj[[i]][[j]]$
        beta_vcov[c(3, 1), c(3, 1)]
      tau_vcov_subj[[i]][[j]] <- result_subj[[i]][[j]]$
        alpha_vcov[c(3, 1), c(3, 1)]
      beta_cook[i,j] <- (1/2) * t(result_all[[i]]$beta -
                               result_subj[[i]][[j]]$beta[c(3, 1)]) %*%
```

```
  solve(as.matrix(beta_vcov_subj[[i]][[j]])) %*%
  (result_all[[i]]$beta - result_subj[[i]][[j]]$beta[c(3, 1)])
beta0_subj[i,j] <- result_subj[[i]][[j]]$beta[3]
beta0_se_subj[i,j] <- result_subj[[i]][[j]]$betaSE[3]
beta1_subj[i,j] <-result_subj[[i]][[j]]$beta[1]
beta1_se_subj[i,j] <- result_subj[[i]][[j]]$betaSE[1]
beta_covratio[i,j] <- det(as.matrix(beta_vcov_subj[[i]][[j]]))/
  det(as.matrix(beta_vcov_all[[i]]))
tau_cook[i,j] <- (1/2) * t(result_all[[i]]$alpha -
                             result_subj[[i]][[j]]$alpha[c(3, 1)]) %*%
  solve(as.matrix(tau_vcov_subj[[i]][[j]])) %*%
  (result_all[[i]]$alpha - result_subj[[i]][[j]]$alpha[c(3, 1)])
tau0_subj[i,j] <-result_subj[[i]][[j]]$alpha[3]
tau0_se_subj[i,j] <- result_subj[[i]][[j]]$alphaSE[3]
tau1_subj[i,j] <- result_subj[[i]][[j]]$alpha[1]
tau1_se_subj[i,j] <- result_subj[[i]][[j]]$alphaSE[1]
tau_covratio[i,j] <- det(as.matrix(tau_vcov_subj[[i]][[j]]))/
  det(as.matrix(tau_vcov_all[[i]]))
sigma_nu_subj[i,j] <- result_subj[[i]][[j]]$sigma_sq_nu
sigma_nu_se_subj[i,j] <- result_subj[[i]][[j]]$sigma_sq_nu_SE
sigma_omega_subj[i,j] <- result_subj[[i]][[j]]$sigma_sq_omega
sigma_omega_se_subj[i,j] <- result_subj[[i]][[j]]$sigma_sq_omega_SE
dfbeta_beta0 <- (beta0_all - beta0_subj)/beta0_se_subj
dfbeta_beta1 <- (beta1_all - beta1_subj)/beta1_se_subj
dfbeta_tau0 <- (tau0_all - tau0_subj)/tau0_se_subj
dfbeta_tau1 <- (tau1_all - tau1_subj)/tau1_se_subj
dfbeta_nu <- (sigma_nu_all - sigma_nu_subj)/sigma_nu_se_subj
```

```
        dfbeta_omega <- (sigma_omega_all - sigma_omega_subj)/sigma_omega_se_subj
      },
      error = function(e){
        warnings(paste("An error occurred for dataset", i,":\n"), e)
      })}
  }
  influence_output <- list(beta_cook = beta_cook, beta_covratio = beta_covratio,
                           tau_cook = tau_cook, tau_covratio = tau_covratio,
                           dfbeta_beta0 = dfbeta_beta0, dfbeta_beta1 = dfbeta_beta1,
                           dfbeta_tau0 = dfbeta_tau0, dfbeta_tau1 = dfbeta_tau1,
                           dfbeta_omega = dfbeta_omega, dfbeta_nu = dfbeta_nu)
  return(influence_output)
}

FastRegLS_influence(result_all = result_all, result_subj = result_subj,
                    nsubj = 50, ndat = 500)
```

References

[1] Gill N, Hedeker D. Fast estimation of mixed-effects location-scale regression models. Statistics in Medicine. 2023;42(9):1430–1444.
